# Large-Scale Land Acquisition and Its Effects on the Water Balance in Investor and Host Countries

**DOI:** 10.1371/journal.pone.0150901

**Published:** 2016-03-04

**Authors:** Thomas Breu, Christoph Bader, Peter Messerli, Andreas Heinimann, Stephan Rist, Sandra Eckert

**Affiliations:** 1 Centre for Development and Environment, University of Bern, Bern, Switzerland; 2 Institute of Geography, University of Bern, Bern, Switzerland; Universidad Veracruzana, MEXICO

## Abstract

This study examines the validity of the assumption that international large-scale land acquisition (LSLA) is motivated by the desire to secure control over water resources, which is commonly referred to as ‘water grabbing’. This assumption was repeatedly expressed in recent years, ascribing the said motivation to the Gulf States in particular. However, it must be considered of hypothetical nature, as the few global studies conducted so far focused primarily on the effects of LSLA on host countries or on trade in virtual water. In this study, we analyse the effects of 475 intended or concluded land deals recorded in the Land Matrix database on the water balance in both host and investor countries. We also examine how these effects relate to water stress and how they contribute to global trade in virtual water. The analysis shows that implementation of the LSLAs in our sample would result in global water savings based on virtual water trade. At the level of individual LSLA host countries, however, water use intensity would increase, particularly in 15 sub-Saharan states. From an investor country perspective, the analysis reveals that countries often suspected of using LSLA to relieve pressure on their domestic water resources—such as China, India, and all Gulf States except Saudi Arabia—invest in agricultural activities abroad that are less water-intensive compared to their average domestic crop production. Conversely, large investor countries such as the United States, Saudi Arabia, Singapore, and Japan are disproportionately externalizing crop water consumption through their international land investments. Statistical analyses also show that host countries with abundant water resources are not per se favoured targets of LSLA. Indeed, further analysis reveals that land investments originating in water-stressed countries have only a weak tendency to target areas with a smaller water risk.

## 1. Introduction

Increased foreign investment in agricultural land has been observed as a key feature of rural transformation in developing countries over the past years, and has become a widely debated topic in the policy and science arenas. Despite numerous articles on the drivers of large-scale land acquisition (LSLA) and its impacts on societies, the economy, and natural resources, the effects of agricultural investments in land on water resources have received only limited attention. Insights from reports and various case studies suggest that foreign investment in agricultural land is substantially motivated by the appropriation of water resources attached to that land [1–3], and some researchers have considered water scarcity in investor countries to be a major driver of investment [2,4]. Irrigation water is viewed as key in making agricultural land investments profitable in the long run [3]. This suggests that water use rights attached to acquired land play a pivotal role in foreign investors’ decisions on where to invest.

Agricultural water consumption worldwide is expected to increase by 70 to 90% over the next 40 years, and by 2025 an estimated two-thirds of the global population will be living in areas experiencing water stress [5]. In light of these projections, water is increasingly regarded as one of the most important limiting factors for future agricultural food and non-food production and is becoming a sought-after commodity for private equity funds [6] and global trade in general. Currently it is estimated that between 16% [7], 22% [8], and 40% [9] of global water consumption is traded as virtual water, which is commonly understood as the water which is embedded in products and used in their production process [9]. Currently 80% of this virtual water is embodied in agricultural commodities, whereas products from the industrial sector account for 20% of global virtual water trade [9]. However, consumption of exterritorial water resources differs substantially between countries. As might be expected, the top importers of foreign virtual water include water-scarce nations in the Mediterranean and the Gulf region, such as Malta (dependency of 92%), Kuwait (90%), Jordan (86%), Israel (82%), the United Arab Emirates (76%), Yemen (76%), Lebanon (73%), and Cyprus (71%). More surprisingly, perhaps, countries with abundant domestic water resources in central and northern Europe, such as Germany, the United Kingdom, and the Netherlands, also rely heavily on external water resources, with dependencies ranging between 60% and 95%. By contrast, many developing countries in sub-Saharan Africa, as well as Argentina and India, import less than 4% of the water they use [8,10].

The increasing globalization of the world’s agricultural sector, not least fuelled by large-scale foreign investments in agricultural land, has a considerable potential for altering local patterns of freshwater use. From a global perspective, trade in agricultural products saved an average 369 Gm^3^/yr of freshwater between 1996 and 2005 [8]. This positive overall effect on the global water balance is achieved mainly by shifting production from countries with high evapotranspiration and low water efficiency rates to countries with a greater water productivity. However, it has been shown that trade in virtual water can exacerbate water stress [7], particularly in exporting countries located in arid and semi-arid regions. In these regions, freshwater resources are likely to decrease as a result of climate change [11]. Reduced availability of freshwater for local production means that local populations have fewer options and a narrower ‘safety margin’ for coping with such climatic extremes [12].

Stress on freshwater resources also exacerbates environmental risks from different forms of land degradation which negatively affect the productive, regulative, supportive, and cultural services of the ecosystems concerned [13,14]. The socio-economic effects of declining freshwater availability, notably in exporting countries of the global South, often directly affect the resilience, livelihoods, and health of the local populations—particularly of the rural poor—and are regarded as a trigger of migration [9]. Moreover, increasing demand for renewable freshwater resources can also fuel domestic water conflicts [15], as access to limited resources is often highly contested; and competition over water may be compounded by pre-existing ethnic, cultural, racial, political, and economic differences. There is consensus, however, that the risk of transboundary water-related conflicts—which are low in number compared to domestic water conflicts—is not directly linked to water scarcity but arises from variously motivated disputes over water allocation between the different riparian states of a given waterbody [16,17]. It is generally assumed that LSLA may exacerbate water conflicts and influence transboundary relations [10,18].

As outlined above, water availability is crucial to the economic viability of investments in land. Many agricultural and non-agricultural uses of land (e.g. mining) depend on the availability of blue water (e.g. rivers, lakes, reservoirs, aquifers) as well as green water (moisture stored in soils and plants) [16]. The acquisition of land includes by default prior rights in green water, as green water is an integral part of the soil. In the case of access and use rights in blue water, experience shows that they either come for free or are included in the investment contracts, which are rarely disclosed to the public. It is widely assumed that investment contracts do not reflect the full value of water. Several researchers engaged in the current land investment debate [3,16,18,19] have argued that investors are not only looking at available land, but that land acquisition is fundamentally about securing water rights.

The assumption that securing water rights is a key motivation for LSLA must be considered of hypothetical nature. To date, only few global studies [4,20,21] have assessed water appropriation in LSLA host countries and its relation to global trade in virtual water. Taking a different approach, we likewise analyse how international investments in agricultural land might alter patterns of freshwater use in host countries, and how they contribute to global trade in agricultural virtual water. In addition, we then go one step further and attempt to examine the validity of the assumption that international LSLA is motivated by the desire to secure control over water resources. To this end, we look at changes in crop water consumption in investors’ countries of origin and in LSLA host countries, taking account of the water demand of crops cultivated on acquired land. Furthermore, we investigate different indicators of investor countries’ motivation to relieve pressure on their water resources, and statistically analyse the interlinkages between host and investor countries against the background of their respective water stress, reflected in the water risk index.

## 2. Materials and Methods

### 2.1 Data on large-scale land acquisitions

Information about specific LSLAs was derived from the Land Matrix database. Since 2009, the Land Matrix initiative has systematically collected and verified information about transnational land investments, and since 2012, it has made this information available to the public via an online data platform [22]. In March 2015, the Land Matrix database contained 955 cases of land transactions recorded since 2000 and covering 200 ha or more; together, these cases covered a total of 30,500 ha of land involved in concluded, intended, and failed land acquisitions, mostly by international investors, through sale, lease, or concession.

This study focuses exclusively on intended and concluded agricultural land deals involving international investors. It does not include investments whose negotiation status is recorded as failed or whose contract was cancelled. Further, we only included cases for which information was available about the area covered, host and investor countries, water risk indices, crop species, crop production per area, and crop water consumption. These restrictions resulted in a sample of 475 land deals, of which 350 are listed as concluded, 60 as intended, and 65 as having an unknown negotiation status. The geographic location of 95 of these deals was known with an accuracy of less than 10 km; of a further 221 deals it was known with an accuracy of 10–100 km; and in the remaining 159 cases only the host country was known. Some of the cases in our sample involved multiple crops, resulting in a total of 862 deal—crop combinations. For the purpose of our calculations, the areas of LSLAs with multiple crops were divided equally by the number of crops specified.

### 2.2 Crop water consumption

The water consumption of each land deal was calculated using crop- and country-specific annual crop production data for 2012 and data on ‘arable land and permanent crop’ for 2011, both retrieved from the FAOSTAT database [23,24], as well as data on water footprints, that is, on the volume of water consumed per unit of crop. Water footprint data were derived from various sources: General water footprints were taken from [25]. Data on flowers were taken from [26]. Data on alfalfa for countries in the Arabian Peninsula were derived from [27]. Country-specific water footprint data for broadleaves, eucalyptus, and pine were taken from [28], which contains water footprint data for different trees, biomes, and countries, as well as country-specific average yields per year. For deals involving trees of unknown species we averaged the water footprint of broadleaves, eucalyptus, and pine. For *Jatropha curcas* we applied the figures for South Africa provided by [29]. Given that no other values were available, we had to use this information for jatropha production in all deals and countries. For all other biofuel crops we were unable to find any suitable water footprint data.

Based on the above datasets, we converted water footprints from m^3^/t to standardized water consumption per unit area (m^3^/ha*year). These standardized country- and crop-specific water footprints were then multiplied with the deal-specific contracted (or, where no contract had been signed yet, intended) areas. Deal-specific water consumption figures were calculated for both investor and host countries, enabling us to determine the total amount of water needed for all deals in each host country, as well as the total amount of water needed in each investor country if the crops grown on land acquired abroad were grown domestically instead. The average annual amount of water used in a host country to produce all crops grown under international land deals (in m^3^/ha*y) and the average annual amount of water used for growing the same crops in the respective investor countries were derived by summing up the water used for every crop type in every land deal and then dividing it by the total area of all land deals located in the host country or originating in the investor country, respectively. The difference between water needed in the host countries of a given investor country’s land acquisitions and water needed in the investor country can be interpreted as the amount of water this investor country saves by shifting production to agricultural land acquired abroad.

In a further step, we derived a water consumption index (WCI) and a water consumption intensity index (WCII) for each host and investor country. The WCI indicates the factor by which the water consumption induced by LSLAs related to a given host or investor country, assuming they were implemented in this country, exceeds this country’s total agricultural water consumption. The WCI of a host country, referred to as WCI_host_, indicates by which percentage the relevant host country’s total agricultural water consumption would increase if all contracted and intended LSLAs in this country were indeed implemented as planned (see S1 File for figures). As such, WCI_host_ gives an indication of how strongly the given host country’s water balance may be affected by the LSLAs it hosts. The WCI of an investor country, referred to as WCI_inv_, shows by which percentage the relevant investor country’s agricultural water consumption would increase if its contracted and intended land deals were implemented domestically rather than abroad (see S2 File for figures). Accordingly, WCI_inv_ provides a hint as to whether the given investor country’s LSLAs might be driven by the desire to relieve stress on domestic water resources. The WCII compares the average crop water consumption per hectare of the studied land deals related to a specific host or investor country, assuming they were implemented in this country, with the total water footprint of this country’s national crop production per hectare. A WCII value greater than 1 indicates that the intensity of water use caused by land deals—assuming they were implemented in the relevant country—exceeds the existing average domestic rate of water use [30]. If the WCII of a host country, referred to as WCII_host_, is greater than 1, this means that the crops planned or implemented in the contracted or intended LSLAs in this host country would put greater stress on its water resources compared to the country’s existing agricultural activities and thus have a disproportionately large negative effect on the national water balance. If the WCII of an investor country, referred to as WCII_inv_, is greater than 1, this indicates that the crops planned or implemented in its agricultural LSLAs abroad would disproportionately stress its own domestic water resources if they were grown domestically; accordingly, a WCII_inv_ greater than 1 can serve as an indicator that the investor country’s LSLAs abroad might be motivated by the desire to reduce pressure on its domestic water resources. The index’s numerator was calculated as the sum of all land-deal-specific water footprints related to a given investor or host country in that country, divided by the total area of the respective land deals. The denominator is the quotient of the total water footprint of national crop production taken from ACQUASTAT [31] divided by the respective country’s total area of arable land and permanent cropland [23]. For example, the WCII_inv_ of Saudi Arabia is 1.3 and was calculated as follows (see S2 and S3 Files for figures): The crops planned or implemented in its reported 25 land deals abroad (totalling 3,764,094 ha) would amount to an average annual crop water consumption of 4,599 m^3^/ha*y if the crops were grown domestically instead; we arrive at the WCII_inv_ if we divide this figure by Saudi Arabia’s current average annual crop water consumption per hectare, which amounts to 3,419 m^3^/ha*y (and is calculated as Saudi Arabia’s total crop water consumption of 11,472 Gm^3^/y divided by its total cropland area of 3,355,000 ha).

### 2.3 Water risk data

Country- and deal-specific agricultural water risk information—expressed in the water risk index (WRI)–is based on the Aqueduct Water Risk Atlas [32]. The WRI is a composite index. It combines twelve water risk indicators to create global maps of water risks that are customizable in terms of indicator weighting, depending on the industry sector being studied. In this study we used the ‘agriculture’ risk weighting scheme. For investor countries’ WRIs, referred to as WRI_inv_, we took the respective country averages, while for host countries’s WRIs, referred to as WRI_host_, we averaged the different land deals’ individual WRIs according to the deals’ geographic locations. Their calculation varied as follows: for deals whose location was known with an accuracy of less than10 km we averaged WRI values within a 10-km radius around the location; for deals whose location was known with an accuracy of 10–100 km we averaged values within a 100-km radius; and for deals whose location information was limited to the host country, we used country averages.

### 2.4 Statistical analysis of water relations between investor and host countries

In a first step, we tested water relations between investor and host countries using three different methods: Pearsons’s correlation coefficient weighted by the area of investment, Spearman’s rank correlation coefficient, and Kendall’s rank correlation coefficient (Tau-b). Cohen’s standard was used to evaluate the correlation coefficient, with coefficients between .10 and .29 representing a small association; coefficients between .30 and .49 a medium association; and coefficients above .50 a large association. In a second step, we performed a standard ordinary least squares (OLS) regression using dummies for the different groups of investor countries.

### 2.5 Limitations

Using the Land Matrix database, national and subnational statistics, and global geospatial datasets presents a number of noteworthy limitations:

- Limitations inherent in the Land Matrix database: [22] and [33] provide a comprehensive overview of challenges related to the Land Matrix data in general. The challenges which are directly relevant to the present study may be summarized as follows: (a) Despite continuous verification, the data in the Land Matrix database might still contain errors due to the heterogeneity of the sources of information. (b) The Land Matrix database is not exhaustive, and its degree of comprehensiveness is geographically biased due to differences in the quality and openness of partnership networks and data providers, as well as a regional unevenness of public and media interest in the phenomenon of LSLA.
- Limitations related to the water footprint calculations: Country-specific national and in some cases subnational water footprint data are available for most food crops that are typically cultivated in a given country. On this basis, our calculations of the water consumption of LSLAs in host countries for food crops may be considered an accurate approximation. Calculation of the amount of water the same crops would consume if grown in the respective LSLAs’ investor countries is more challenging. Many crops grown on land acquired abroad are not cultivated at all in the relevant investor countries, and respective water footprint data may therefore not exist. In such cases, we approximated water footprint data using either regional averages from neighbouring countries or values for countries with similar agroecological conditions. In few cases where water footprint data were not available for any similar agroecological zone, we used crop-specific global averages. A specific case in point is the crop water consumption of *Jatropha curcas*, for which we were unable to find any country-specific data. As a rough approximation we used the water consumption value for jatropha in South Africa provided by [29]. All other water footprint data for biofuels refer to the production of jatropha oil [34].

## 3. Results

### 3.1 Large-scale land acquisition and the global water balance

As shown in Table 1, if all LSLAs considered in this study were actually implemented, the 475 projects would consume 91.9 Gm^3^ of water annually on an area of 26,392,014 ha, which is nearly the size of New Zealand. Disaggregated data on green, blue, and grey water consumption were available for 77.2 Gm^3^/y of the water required by our sample of land deals. Of this amount, 73% is green water, 8% is blue water, and 3% is grey water. For the remaining 14.7 Gm^3^/y or 16% of the water required by our sample of land deals only the global water footprint average was available (e.g. for *Jatropha curcas* and *Pongamia pinnata*).

**Table 1:** Large-scale land acquisitions (LSLAs) and their contribution to the global water balance by origin of investors.

Investors’ region of origin	Number of land deals	Contracted or intended LSLA area [ha]	Water consumption of LSLAs in host countries [Gm^3^/y]	Water consumption if LSLA crops were produced domestically in investor countries [Gm^3^/y]	Contribution to global water savings [Gm^3^/y]
**Asia**	261	16,209,377	55.6	79.0	23.4
**Africa**	42	2,698,258	9.5	12.1	2.6
**South America**	15	1,546,956	5.9	2.7	-3.2
**North America**	49	2,633,264	9.4	21.2	11.8
**Europe**	102	2,476,477	9.3	7.0	-2.2
**Oceania**	6	827,682	2.2	1.9	-0.3
**Total**	475	26,392,014	91.9	123.9	32.1

If we assume that agricultural commodities from LSLAs are produced for export only, they would increase the average annual international virtual water flow through agricultural products for 1996 to 2005 of 1597 Gm^3^/y [8] by up to 5.8% (corresponding to the above 91.9 Gm^3^/y). If all crops were produced domestically in the respective investor countries, the water requirements would amount to 124.0 Gm^3^/y, surpassing the total water consumption for production in the designated host countries by 32.1 Gm^3^/y or 23.4%. Compared to the global water savings through virtual water flows of 369 Gm^3^/y [8], the analysed LSLAs have the potential to further increase water savings through virtual water trade by 8.7%. Analysis by continent (according to the UN nations geoscheme classification) shows that, on average, LSLAs by Asian (including the Gulf States), North American, and African investors would contribute to global water savings, whereas LSLAs by European, South American, and Oceanian investors would have an adverse effect on the global water balance. However, country aggregation by continents significantly blurs the actual contributions of the different investor countries. Out of 54 investor countries, 22 countries would relieve the global water balance by 44.8 Gm^3^/y by implementing their land deals, whereas 34 investor countries would add 12.7 Gm^3^/y to global water consumption for agricultural production. The investor countries that would contribute most to global water savings by externalizing the production of agricultural commodities are the United States (12.5 Gm^3^/y), Singapore (9.2 Gm^3^/y), Saudi Arabia (7.3 Gm^3^/y), Japan (4.4 Gm^3^/y), Zimbabwe (3.5 Gm^3^/y), the United Arabian Emirates (2.9 Gm^3^/y), India (2.3 Gm^3^/y), and China (1.3 Gm^3^/y). Investor countries where agricultural production would require less water at home and whose LSLAs would hence worsen the global water balance include Malaysia (-3.2 Gm^3^/y), Brazil (-2.9 Gm^3^/y), Canada (-1.8 Gm^3^/y), as well as 12 European countries (totalling -2.2 Gm^3^/y).

### 3.2 Water balance effects of large-scale land acquisition in host countries

The water consumption of crop production in the analysed LSLAs relates to a total of 59 host countries. Of the total water consumption by our sample of land deals, 88.5% would occur in lower-middle-income, low-income, and least-developed countries (classification according to the DAC list of ODA recipients for 2012 and 2013 reporting). From a regional perspective, 54.4% of the water consumption would occur in Africa, 35.3% in Asia, 9.1% in South America, and 1.2% in Europe and Oceania together. Zooming in on individual countries, it is worth noting that a large share of this water consumption would be concentrated in relatively few host countries: Almost 65% of the total crop water consumption by LSLAs would occur in only ten host countries (see S1 File). Among these, the five sub-Saharan states of Ethiopia, Mozambique, Sudan, South Sudan, and Sierra Leone would account for almost 30.6% of the total LSLA-induced water consumption, while the four tropical Asian countries of Indonesia, the Philippines, Cambodia, and Laos would account for another 30.4%. Brazil, also among the top ten, would accommodate 3.6% of water consumption.

To get an indication of the potential effects of LSLA on the current water resource situation in host countries, we calculated the water consumption index (WCI) for host countries. WCI_host_ compares the water consumption of the planned crops in all contracted or intended LSLAs in a given host country to that of the country’s current total agricultural production. On average, the analysed land deals would require 1.8% of the current agricultural water consumption in host countries. Out of the 59 countries analysed, 21 –including 17 sub-Saharan countries—would see an increase greater than 5% of their current total agricultural water consumption if all LSLAs targeting these countries were implemented. Prominent examples of countries that would be affected by a significant increase in water consumption are the Republic of the Congo, where implementation of all LSLAs in our sample would increase water consumption by a stunning 181%, as well as Sierra Leone (76%), Laos (52%), Uruguay (45%), South Sudan (36%), and Cambodia (33%) (see WCI_host_ in S1 File).

The above figures are only of indicative nature, as they do not reflect substitution effects where LSLAs would replace prior agricultural land uses, and also given that the Land Matrix data are incomplete by default. To further substantiate the potential effects of LSLAs on water resources in host countries, we applied the water consumption intensity index (WCII) to host countries. WCII_host_ compares average crop water consumption per hectare of planned and implemented agricultural production on land acquired by foreign investors in a given host country with the existing national average crop water consumption per hectare in that host country. Of 59 countries analysed, 21 countries (LSLA area share of 36.0% and total water consumption share of 50.1%) would experience an increased intensity of water use per hectare compared to their current average agricultural production. The increase in water consumption intensity would be most prominent in Cambodia, where LSLAs would consume more than double as much water per hectare compared to the country’s current average agricultural water consumption. States with substantial areas affected by LSLA, such as Sudan and Ethiopia, would experience an increase by 64% and 60%, respectively, compared to their current agricultural water consumption per hectare. In this regard, as well, sub-Saharan states are disproportionately affected, with 15 countries in this region experiencing an above-average water consumption intensity (WCII_host_ > 1) of LSLAs compared to their current average agricultural water consumption (see WCII_host_ in S1 File).

To determine whether LSLAs target host countries with abundant water resources or whether they affect regions already experiencing water stress, we compared host countries’ water consumption and water consumption intensity indices (WCI_host_ and WCII_host_) with their water risk index (WRI_host_) as an indicator for water stress. Spearman’s rank correlation between WCI_host_ and WRI_host_ is -0.26 (p = 0.052); between WCII_host_ and WRI_host_ it is -0.27 (p = 0.039). Pearson’s correlation coefficient, which is area-weighted, shows a similar picture, with a value of -0.18 (p = 0.163) between WCI_host_ and WRI_host_, and -0.46 (p = 0.001) between WCII_host_ and WRI_host_. Both correlation coefficients characterize the correlation between WCI_host_ and WRI_host_ as not significant, whereas WCII_host_ shows a significant inverse relation with WRI_host_. Based on these results, we can conclude that host countries with abundant water resources are not per se favoured as target areas of transnational investments in agricultural land. However, LSLAs for agricultural activities of above-average water intensity tend to target host countries with a relatively low water stress.

### 3.3 Investor countries’ contributions to water consumption linked to large-scale land acquisition

Table 2 shows that in terms of the origins of investments, 33.4% of the above water balance effects in host countries are related to LSLAs by investors from high-income countries, followed by the BRICS states (23.6%) and upper-middle-income developing countries (19.2%), whereas the Gulf States account for 16.3%, and lower-middle-income, low-income, and least-developed countries together are responsible for only 7.5% of the total LSLA-related water consumption. Water consumption per hectare if LSLA crops were grown domestically in the investor countries differs considerably between the categories; water consumption intensity would be lowest in the BRICS states and highest in the high-income countries. Much of the total LSLA-related water consumption in host countries is linked to relatively few investor countries: out of all 54 investor countries considered, 6 account for more than half of the total LSLA-related water consumption in all host countries. The list of top water-consuming investor countries is led by Saudi Arabia and China, which each account for 10.9% of the overall LSLA-related water consumption, followed by Malaysia (10%), the United States (8.1%), India (6.8%), and Brazil (5.2%) (see S2 File).

**Table 2:** Water consumption of large-scale land acquisitions (LSLAs) by investor country category.

Investor country category	Number of land deals	Contracted or intended LSLA area [ha]	Water consumption of LSLAs in host countries [m^3^]	Water consumption if LSLA crops were produced domestically in investor countries [m^3^]	Share of total LSLA-related water consumption in host countries	Water consumption per unit area in host countries [m^3^/ha]	Water consumption per unit area if LSLA crops were produced domestically in investor countries [m^3^/ha]
**High-income countries**	210	9,118,877	30,723,164,594	53,505,812,148	33.4%	3,369	5,868
**Gulf States**	46	5,301,787	14,993,187,716	25,224,997,304	16.3%	2,828	4,758
**BRICS countries**	113	6,208,873	21,644,429,509	22,643,702,944	23.6%	3,486	3,647
**Upper-middle-income countries**	76	3,646,400	17,621,319,223	14,235,125,220	19.2%	4,833	3,904
**Lower-middle-income, low-income, and least-developed countries**	30	2,116,077	6,934,602,083	8,369,194,006	7.5%	3,277	3,955
**Total**	475	26,392,014	91,916,703,124	123,978,831,622	100%	3,483	4,698

### 3.4 Approximating the water balance effects of large-scale land acquisition in investor countries

The few existing global studies on the water balance effects of LSLA—which has also been referred to as ‘water grabbing’–focused mainly on effects in host countries. However, to get an indication of whether land acquisition is indeed motivated by the desire to secure water resources and shift water consumption abroad in order to relieve pressure on domestic water resources, we need to examine the effects of LSLA on water resources in investor countries.

To get a first hint at the country level whether investors’ motivation might be to appropriate foreign water resources, we calculated the water consumption index (WCI) for investor countries. WCI_inv_ shows by which percentage agricultural water consumption in a given investor country would increase if its contracted and intended land deals were implemented domestically. The analysis shows that several Gulf States, such as Saudi Arabia, the United Arab Emirates, Qatar, Kuwait, and Bahrain, as well as Brunei, Singapore, Djibouti, the Cayman Islands, and Cyprus would experience the most pronounced increases in their total agricultural water consumption if they were to produce the crops outsourced through land investments domestically instead. Disregarding any substitution effects, Saudi Arabia, for instance, by growing all crops at home would increase its total agricultural water consumption by 51%, while the other Gulf States—not least as a result of comparatively limited agricultural land areas—would experience an even greater increase between 181% (United Arab Emirates) and 1113% (Qatar) in their total agricultural water consumption (see WCI_inv_ in S2 File).

A better indication of whether investor countries’ motivation is to reduce pressure on their own water resources can be gained by looking at their water consumption intensity index (WCII_inv_). This index compares average crop water consumption per hectare of an investor country’s contracted and intended LSLAs if the crops were grown domestically in the investor country with that country’s current average agricultural crop consumption per hectare. Of the 54 investor countries analysed, 20 countries have land deals whose water consumption intensity, if the deals were implemented domestically, would exceed the respective countries’ current average agricultural water consumption per hectare (see WCII_inv_ in S2 File). These countries are disproportionately externalizing production of water-intensive agricultural goods to foreign countries. Among them are big investor countries such as the United States, whose land deals are 79% more water-intensive compared to the existing average crop water demand per hectare (WCII_inv_ = 1.79), as well as Saudi Arabia (1.35), Singapore (3.55), and Japan (2.07). By contrast, several prominent investor countries who have repeatedly been suspected of seeking to secure water resources have land deals that would actually require below-average amounts of water if implemented domestically. China, for example, would actually use only 48% of its current average water consumption per hectare to grow domestically the crops it produces abroad under land deals (WCII_inv_ = 0.48). Similarly, India (0.85) and all Gulf States other than Saudi Arabia (0.16 to 0.89) invest in agricultural activities abroad that are less water-intensive compared to their average domestic crop production. This analysis shows that relatively few countries are acquiring land abroad to grow crops that consume above-average amounts of water in domestic production—although these few countries account for a large share in the total average annual water consumption of all land deals. A majority of the investor countries have land deals whose crops, if grown domestically, would require less water than the respective investor country’s current average agricultural production.

### 3.5 Analysing the interlinkages between investor and host countries

Various authors argue that water scarcity in investor countries and displacement of agricultural water demands must be seen as a major driver of international water flows [1,18,30,35]. Especially investor countries such as India and China [36] and limited natural water resources in the Gulf States [37] were cited in support of this argument. There is a general consensus that rich and reliable water resources and the potential for irrigation are key criteria in the selection of land abroad for investment [3,38].

**Fig 1:**
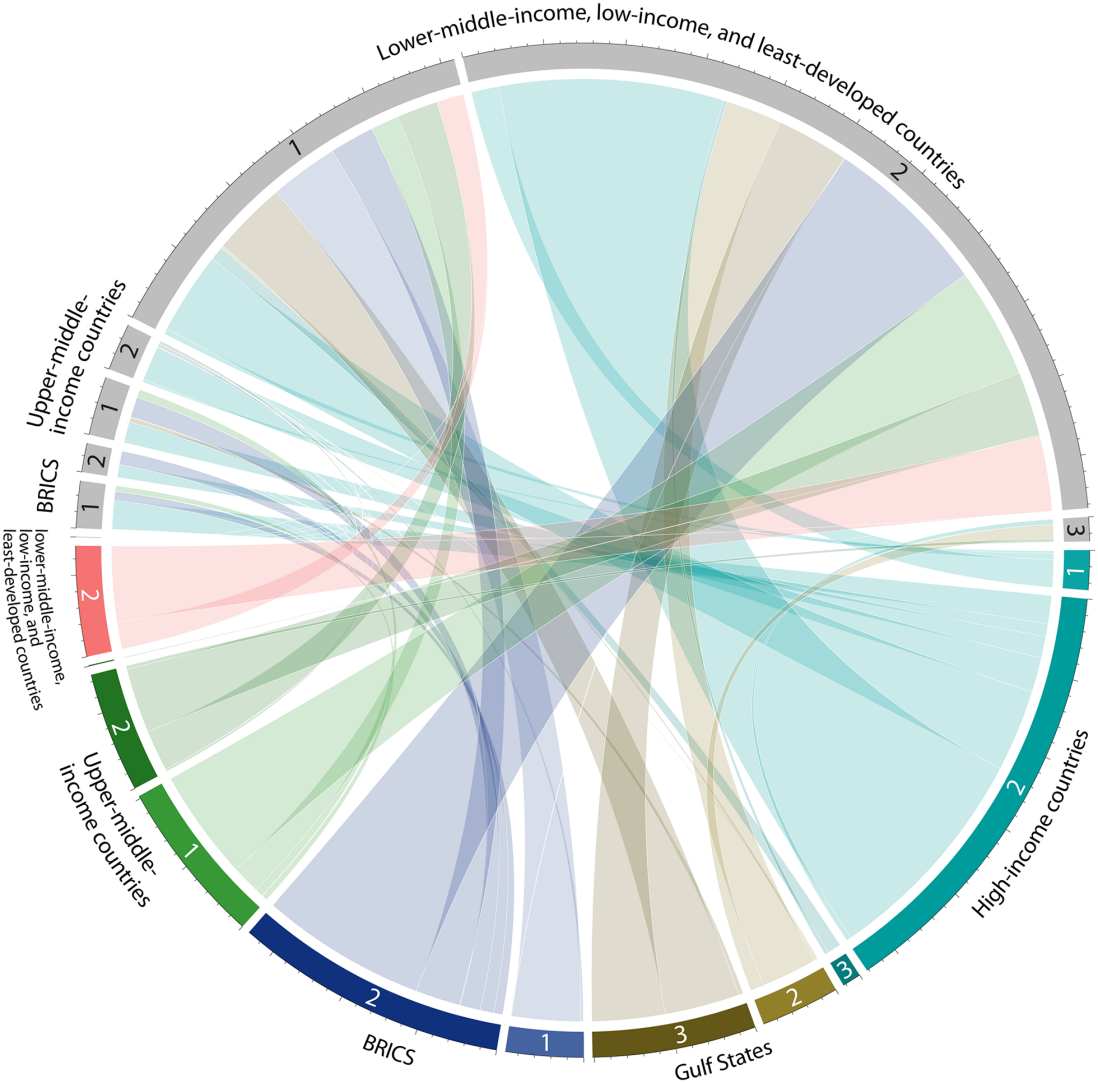
LSLA-induced water flows between investor and host country categories differentiated by water risk. This graph depicts water flows induced by the analysed land deals between investor countries (coloured) and host countries (grey). The size of the coloured bands represents the amount of water used (Gm^3^) under agricultural land deals in a given category of host countries by a given category of investor countries. Based on the water risk indices (WRIs) of investor countries, we defined three subcategories: (1) low water risk (WRI of 0 to 1.5), (2) medium water risk (WRI of 1.5 to 3), (3) high water risk (WRI greater than 3).

Almost 88% of the analysed LSLAs’ total water consumption happens in lower-middle-income, low-income, and least-developed countries. Looking at water stress, 66% occurs in areas experiencing medium water stress (WRI of 1.5 to 3), 32% in areas with no or little water stress (WRI of 0 to 1.5), and 2% is in areas under high water stress (WRI greater than 3). A closer look at water flows between investor and host country categories (Fig 1) reveals that BRICS countries invest almost exclusively in developing host countries with water risks comparable to their own. The Gulf States show a more differentiated picture: Gulf countries with a high water risk cause LSLA-related water consumption mainly, and in equal shares, in lower-middle-income, low-income, and least-developed countries with low and medium water risks, while Gulf countries with a medium water risk index account for LSLA-related water consumption primarily in host countries with a medium water risk and, to a lesser degree, in those with a high water risk. High-income countries are responsible for 26.2% of LSLA-related water consumption in lower-middle-income, low-income, and least-developed countries, but also account for 7.2% of total LSLA-induced water consumption occurring outside this country category. Almost half of the water consumption induced by high-income countries’ investments occurs in lower-middle-income, low-income, and least-developed countries with a medium water risk index, while 17.7% occurs in countries with a low water risk and hence abundant water resources. This descriptive analysis reveals distinct investment preferences. Generally land investments tend to link investor countries and host countries with similar water risk situations. Nonetheless, we also observe a certain tendency among some investor country categories, such as high-income countries, water-stressed Gulf States, and upper-middle-income developing countries, to prefer areas with a low water risk.

In the following, we present a statistical analysis of whether LSLAs by water-stressed investor countries disproportionately target areas with abundant water resources and thus with a WRI lower than that of the investor country. The result of the area-weighted regression analysis of 475 land deals shows a negative correlation, with a coefficient of -0.14 (p < 0.012) and no statistical anomalies in the 95% confidence interval of the five country categories analysed. In other words, investor countries show a general tendency, although rather weak, to invest in host countries with a smaller WRI and thus with more abundant water resources (see S1 Table). Looking at the different country categories, we found a significant negative correlation for investments by the Gulf States and upper-middle-income countries, which means both categories largely invest in countries with a lower WRI compared to their own. By contrast, the correlation was positive for investments by the BRICS countries. No statistically significant correlation was found for investments by high-income countries and lower-middle-income and least-developed countries (see S2 Table).

**Fig 2:**
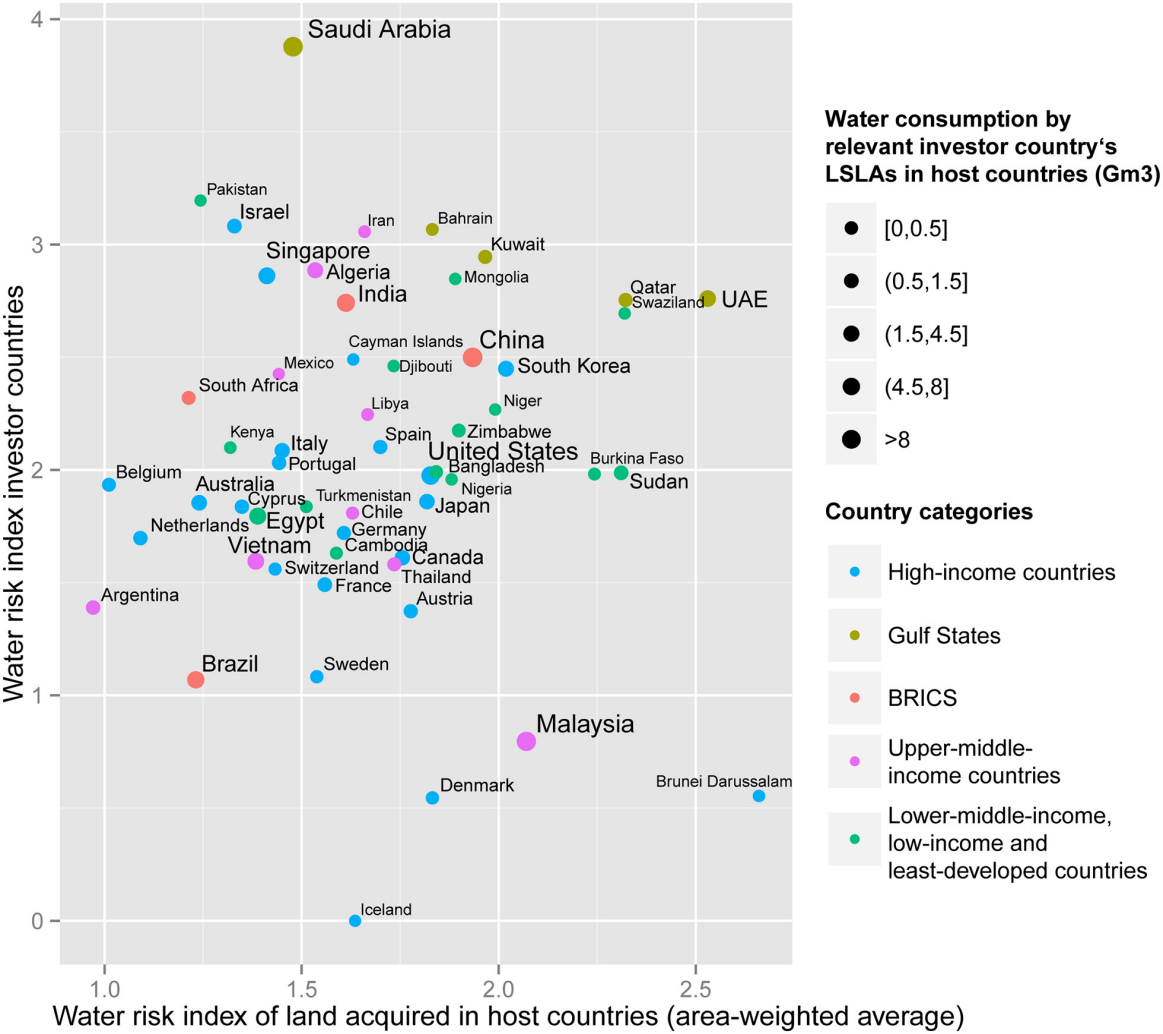
Investor countries by domestic water risk and area-weighted average water risk of land they acquired in host countries.

Given that the above analysis is based on an aggregation of different countries, neutralization effects within each country category are likely. For this reason, we took a closer look at the country level and found four main types of investor country (Fig 2). The first group comprises investor countries experiencing above-average domestic water stress who prefer to invest in host countries with little water stress and thus more abundant water resources. This group includes some prominent investor countries known for their water stress, such as Saudi Arabia, China, India, Israel, and Algeria. However, it also includes countries with a prominent financial sector, such as Singapore and Cayman Islands. This group probably corresponds best to the notion that domestic water shortages are the primary motivation for externalizing agricultural production, and thus water use. The second and largest group comprises investor countries with comparatively little domestic water stress (WRI < 2) who invest in host countries with a similar water risk. This group seems to be interested in appropriating land per se and not necessarily as a means of relieving pressure on domestic water resources. The group contains many high-income countries, as well as some investor countries in Latin America (Brazil, Argentina, and Chile) and Southeast Asia (Thailand and Cambodia). A third group comprises a small number of water-stressed countries (WRI > 2) such as Qatar, the United Arab Emirates, and Swaziland with land investments in fairly water-stressed countries. An underlying reason for investments in countries with similar water resources might be that the crops to be produced to meet domestic market demands grow best under similar biophysical conditions. The fourth group consists of only two investor countries, Malaysia and Brunei, who have a low domestic water risk (WRI < 2) but invest in countries with an above-average water risk.

## 4. Discussion and Conclusions

As outlined in the introduction, the overarching objective of this paper is to challenge the narrative that investor countries’ key motivation for acquiring large stretches of agricultural land abroad is to secure water rights and relieve pressure on their domestic water resources. Besides analysing how intended and concluded LSLAs documented in the Land Matrix database affect the water balance in host countries and how they contribute to global trade in virtual water, this study examined how crop water consumption in investor countries would change if their LSLAs were implemented domestically. In addition, we did a statistical analysis of LSLA-related interlinkages between host and investor countries, comparing their respective levels of water stress. The following section summarizes and discusses the main findings of this study, outlines possible policy implications, and suggests considerations for future research.

### 4.1 Main findings

This study shows that implementation of all 475 LSLAs analysed would result in an annual crop water consumption of 91.9 Gm^3^, corresponding to 1.8% of the current agricultural water consumption in host countries. Under the assumption that commodities from LSLAs are produced for export only, this indicates a further increase in global water savings through virtual water flows by 8.7%. Nearly two-thirds of crop water consumption by LSLAs would be concentrated in only 10 out of 59 host countries; half of the most affected countries are located in sub-Saharan Africa. In at least 21 host countries, crop water consumption per hectare would increase compared to their average current agricultural water consumption; these countries likewise include 15 sub-Saharan states that would be affected by above-average water consumption intensities as a result of LSLA. Statistical analysis revealed that host countries with abundant water resources are not per se preferred to arid or semi-arid countries as target areas of LSLA, but that water-intensive LSLAs tend to target host countries with a relatively low water risk. These findings suggest that several host countries face the risk of LSLA increasing pressure on the local water balance. Particularly in arid and semi-arid areas, such increased pressure is likely to intensify competition over water at the expense of local land use systems.

The study further reveals that a small number of investor countries are responsible for a large share of LSLA-related water consumption in the targeted host countries: as few as 6 out of 54 investor countries—Saudi Arabia, China, Malaysia, the United States, India, and Brazil—account for more than half of the total LSLA-related water consumption in host countries. We also show that LSLAs by 20 investor countries would increase these countries’ average domestic crop water consumption if they were implemented on their home soils, indicating that these countries’ investments in land abroad might indeed be motivated by the intention to reduce pressure on their own water resources. This group of countries who are disproportionately externalizing crop water consumption include big investor countries such as the United States, Saudi Arabia, Singapore, and Japan. At the same time, several countries often suspected of acquiring land abroad to relieve pressure on their domestic water resources—such as China, India, and all Gulf States except Saudi Arabia—invest in agricultural activities abroad that are less water-intensive compared to their average domestic crop production. In view of this finding, we cannot confirm the hypothesis that these countries’ investments in land abroad are primarily motivated by the intention to relieve pressure on their domestic water resources. The exception among the Gulf States is Saudi Arabia, whose LSLA strategy probably reflects recently introduced national policies for water savings in agriculture.

A regression analysis across all 475 land deals in our sample showed only a weak general tendency of water-stressed investor countries to disproportionately target host countries with abundant water resources. When looking at the different country categories, however, we found a statistically significant tendency of Gulf States and upper-middle-income countries to acquire land in countries that face a water risk lower than their own, while South American investor countries tend to target areas facing a water risk higher than their own. However, this statistical relation primarily reflects climatic conditions in the respective investor countries. The Gulf States have an arid to semi-arid climate and a high water risk, whereas South American investor countries have a humid to temperate climate and a low water risk. Given the climatic zones to which these two country groups belong, they are almost forced to invest in areas with a water risk different from their own. Accordingly, this tendency cannot per se be seen as reflecting a primarily water-related motivation for acquiring land abroad.

### 4.2 Policy considerations

This study has clearly shown that the effects of LSLA on water resources risk to further aggravate competition over water in a number of host countries. This is highly relevant, as water crises are considered to be the most relevant global risk in terms of impact [39]. Further, there are clear indications that a relatively small number of large investor countries are disproportionately relieving pressure on their own water resources by acquiring land abroad. Based on our findings, two main policy arenas should be considered. A first major policy challenge is to find appropriate governance mechanisms for investments in agricultural land. These mechanisms must be able to deal with the specificities of water as a resource, which is by nature variable over time and moves across administrative levels, political boundaries, and biophysical contexts. In recent years, numerous global and regional initiatives developed tools and guidelines on foreign investments in land to support governments in host countries. These tools and guidelines provide important principles to ensure responsible investments, as well as benchmarks to test investment contracts. However, a general flaw of these tools is that they are of voluntary nature and often include no provisions for monitoring and reporting [40]. A further commonality of these initiatives is that they do not, or only indirectly, address the water issues related to LSLA, and that they do not consider the specific characteristics that distinguish water from other natural resources. From a local perspective, adequate regulatory or legal provisions for guiding investments and for ensuring availability of and access to water for other user groups at different geographical locations are often lacking. A promising way of tackling LSLA-related water issues is offered by the principle of subsidiarity, which requires that policies and instruments at local and basin scales are complemented with regional and global regulations and binding guidelines on investment in agriculture, including specific provisions for water-related issues.

A second important focus of policies to mitigate water-related risks of LSLA concerns sustainable land and water management practices. Water management technologies such as crop rotation, mulching, minimum till, floodwater harvesting, and rainwater trapping offer opportunities to increase the availability of blue and green water [40,41] and to limit potential off-site effects of LSLAs on land and water elsewhere. To reduce evaporation and surface runoff, local policies on LSLA should promote locally adapted improved water and land management practices and inclusion of corresponding provisions in LSLA contracts.

### 4.3 Conceptual and methodological issues

This study aimed at examining the assumption that international LSLA is motivated by the desire to secure control over water resources—an assumption variously made in recent reports and scientific articles under the general heading of ‘water grabbing’. The study does not, however, claim to assess the extent of water resource appropriation in an absolute manner (see limitations in section 2.5). The aim was rather to shed light on various patterns and interlinkages characterizing the effects of LSLA on water resources. In order for future global studies on the water-related effects of LSLA to generate more precise insights, basic input data on LSLAs must improve in terms of completeness and resolution. A major shortcoming of currently available data on LSLAs—particularly when aiming to study their effects on water resources—is the widespread lack of information about prior land uses and land covers. Only if this information becomes available can substitution effects be factored in and the effects of LSLA on water balances be estimated closer to reality. Improvements are also needed with regard to information on crop water consumption. For several crop species this information either does not exist at all, or is not differentiated in terms of agroecological zones; this is particularly true of water consumption figures for biofuel crops.

However, even if we achieve more accurate estimations of the aggregated effects of LSLA on water resources and their interlinkages, this will not automatically lead to a better understanding of the underlying processes or to the ready identification of opportunities for a better stewardship of LSLAs in general and regarding their water effects in particular. Generating this type of knowledge requires in-depth case studies which take into account socio-ecological settings and explicitly integrate the water-related aspects of LSLAs. Meta-analyses of these case studies will then be needed to generalize research results, to quantify and qualify water-related impacts of LSLA on society, ecology, and the economy, and to identify key factors and processes involved.

## Supporting Information

S1 File Water consumption induced by large-scale land acquisitions (LSLAs), by host country. This Excel file contains various key figures characterizing water consumption induced by the 475 large-scale land acquisitions examined in this paper, as well as the water risk index, for each of the 59 host countries.
(XLSX)

S2 File Water consumption induced by large-scale land acquisitions (LSLAs), by investor country. This Excel file contains various key figures characterizing water consumption induced by the 475 large-scale land acquisitions examined in this paper, as well as the water risk index, for each of the 54 investor countries.
(XLSX)

S3 File Supplementary data on large-scale land acquisition and its effects on the water balance. This Excel file contains key figures characterizing the 475 large-scale land acquisitions analysed in this paper and their water consumption.
(XLSX)

S1 Table Regression analysis for the water risk indices (WRIs) of investor and host country categories. This file contains a table showing statistical data for the regression analysis of the WRIs of investor country categories (WRI_inv_) and host country categories (WRI_host_).
(XLSX)

S2 Table Pearson’s correlation coefficient for the water risk indices (WRIs) of investor and host country categories. This file contains a table showing Pearson’s correlation coefficients for the WRIs of investor country categories (WRI_inv_) and host country categories (WRI_host_), weighted by the area of investment.
(XLSX)
